# Ascending Colon Schwannoma: A Rare Submucosal Tumour

**DOI:** 10.7759/cureus.54902

**Published:** 2024-02-25

**Authors:** Kugendran Ponniah, Larissa Yong, Abdul Rana, Christopher McDonald

**Affiliations:** 1 Department of Surgery, Northern Adelaide Local Health Network, Adelaide, AUS

**Keywords:** immunohistochemistry, histology, diagnosis, ascending colon, schwannoma

## Abstract

We present a rare case of schwannoma in the ascending colon of a 60-year-old female with dyslipidemia. A series of diagnostic procedures, including colonoscopy and CT colonography, led to the successful robotic-assisted right hemicolectomy. Histological and immunohistochemical analyses confirmed the diagnosis of schwannoma, and the patient achieved a complete recovery post-surgery.

## Introduction

Schwannomas, though infrequent in the gastrointestinal tract, can present diverse clinical challenges. Our case highlights a unique manifestation of schwannoma in the ascending colon, an unusual location for this type of tumour [1]. Through a combination of diagnostic investigations and surgical intervention, we navigated the challenges posed by this condition, emphasizing the significance of accurate diagnosis and appropriate management strategies.

## Case presentation

We present the case of a 60-year-old female with a background of dyslipidaemia, referred by her general practitioner after a positive fecal occult blood test (FOBT). The patient reported experiencing intermittent diarrhea over the past year, accompanied by an unintentional weight loss of 5 kg. Notably, she denied any abdominal pain or rectal bleeding but had a family history of bowel cancer at 80 years of age.

Thus, we proceeded with a colonoscopy which revealed a 35 mm submucosal polypoidal lesion in the ascending colon, and subsequent biopsies confirmed the benign nature of the lesion, although submucosa evaluation was not possible due to the sampling limitation.

**Figure 1:**
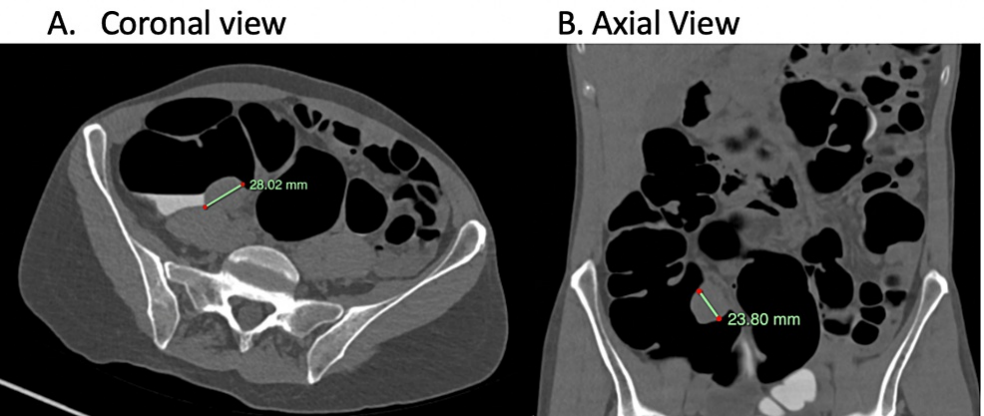
CT colonography. A 28.02 x 23.8 mm circumscribed ovoid soft tissue lesion on the posterior wall of the caecum

Subsequently, a CT colonography was done that demonstrated a 28 x 23.8 mm circumscribed ovoid soft tissue lesion on the posterior wall of the caecum, proximal to the ileocecal (IC) valve, with no evident infiltration of the adjacent pericolic fat, except for a small (5 mm) non-specific pericolic node (Figure 1). Given the differential possibilities, including a gastrointestinal stromal tumour (GIST), leiomyoma, and lymphoproliferative lesions, a robotic-assisted right hemicolectomy with intracorporeal anastomosis was performed.

**Figure 2:**
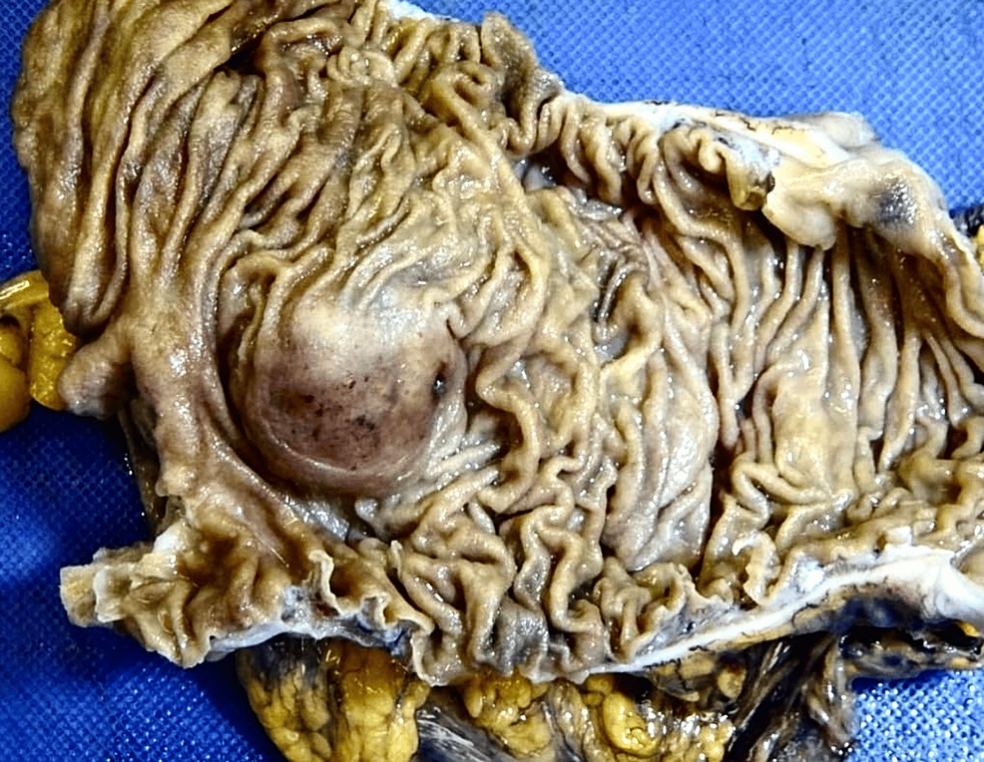
A 34 x 31 x 17 mm submucosal nodule

**Figure 3:**
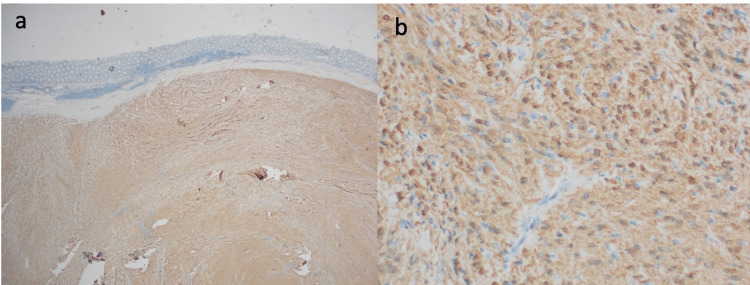
A) H&E stain showing spindle to ovoid cells with a diverse cellular pattern. (B) Tumor immunohistochemistry (IHC) stain for S100 proteins

Macroscopically, the ascending colon mucosa exhibited a 34 x 31 x 17 mm submucosal nodule, accompanied by discoloured mucosa (Figure 2). Upon a histological examination, the findings revealed a submucosal proliferation of spindle to ovoid cells with a diverse cellular pattern, along with an associated chronic inflammatory response. This response included the presence of a rich lymphoid cuff, lymphocytes, plasma cells, and a sparse population of mast cells. Additionally, vessels with hyalinized walls were observed, and the lesion appeared to have invaded the muscularis propria. The immunohistochemical analysis displayed diffuse and strong nuclear positivity for S100 proteins, confirming the diagnosis of schwannoma (Figure 3).

The post-operative histology indicated a complete resection, and no further treatment was deemed necessary. The patient had an uneventful recovery and was followed up in the clinic after two weeks to discuss the benign nature of the histology report.

## Discussion

Schwannomas, a relatively rare type of peripheral nerve sheath tumour in the gastrointestinal (GI) tract, represent a small fraction (2-6%) of all mesenchymal tumours, which suggests their infrequency in clinical practice [1,2]. While GI schwannomas are uncommon, their occurrence in the ascending colon is exceptionally rare, with previous literature emphasizing the rectum as the most prevalent location for benign schwannomas, followed by the right colon [3,4]. Additionally, the slightly higher prevalence among females and their average manifestation during the sixth decade of life highlight the specific demographic trends associated with this rare pathology [5].

Despite their usual asymptomatic progression, some GI schwannomas present with pain, tenesmus, and bleeding, warranting meticulous diagnostic approaches and timely interventions [6,7]. Schwannomas are often found incidentally during regular colonoscopy surveillance or CT scans done for other reasons. They seldom erode the mucosa, typically appearing as submucosal masses or polyps during colonoscopy or CT scans [7,8].

An accurate diagnosis relies on the immunohistopathological examination of the surgically removed specimen. When observed macroscopically, these tumours typically manifest as distinct, well-defined masses with several lobulated areas, occasionally exhibiting mucosal ulceration. Schwannomas exhibit positive S100 staining while they display negative staining for various markers, including DOG1, SMA, desmin, CD 117, CD-34, and c-KIT, aiding in their differentiation from other mesenchymal cells [9,10].

Although schwannomas generally exhibit a benign and indolent clinical course, the potential for incomplete excision leading to recurrence and rare instances of malignant transformation emphasize the necessity for comprehensive surgical resection with negative margins [11].

Attaining complete surgical resection with tumour-free margins is considered the most effective therapeutic strategy. Incomplete surgical resection and insufficient margins are often implicated in tumour recurrence. Adjuvant therapies are generally not recommended when negative margins are successfully achieved through surgery [12].

## Conclusions

In cases where a growth is seen during colonoscopy, accurately diagnosing schwannomas through biopsy poses significant challenges and carries the risk of a missed diagnosis. Consequently, surgical intervention becomes imperative to definitively determine the benign nature of the growth and ensure that the tumour is completely excised to prevent a recurrence. In addition to that, a clear examination of the removed tissue under a microscope, especially looking for S100, is crucial for confirming the diagnosis of schwannomas. Given the benign nature of this pathology, a complete resection would be adequate without further need for chemotherapy and radiotherapy post surgery.

## References

[1] Mekras A, Krenn V, Perrakis A (2018). Gastrointestinal schwannomas: a rare but important differential diagnosis of mesenchymal tumors of gastrointestinal tract. BMC Surg.

[2] Bohlok A, El Khoury M, Bormans A (2018). Schwannoma of the colon and rectum: a systematic literature review. World J Surg Oncol.

[3] Nonose R, Lahan AY, Santos Valenciano J, Martinez CA (2009). Schwannoma of the colon. Case Rep Gastroenterol.

[4] Inagawa S, Hori M, Shimazaki J (2001). Solitary schwannoma of the colon: report of two cases. Surg Today.

[5] Kanneganti K, Patel H, Niazi M, Kumbum K, Balar B (2011). Cecal schwannoma: a rare cause of gastrointestinal bleeding in a young woman with a review of literature. Gastroenterol Res Pract.

[6] Tsunoda C, Kato H, Sakamoto T (2009). A case of benign schwannoma of the transverse colon with granulation tissue. Case Rep Gastroenterol.

[7] Skopelitou AS, Mylonakis EP, Charchanti AV, Kappas AM (1998). Cellular neurilemoma (schwannoma) of the descending colon mimicking carcinoma: report of a case. Dis Colon Rectum.

[8] Tanaka T, Ishihara Y, Takabayashi N, Kobayashi R, Hiramatsu T, Kuriki K (2011). Gastrointestinal: asymptomatic colonic schwannoma in an elderly woman; a rare case. J Gastroenterol Hepatol.

[9] Çakır T, Aslaner A, Yaz M, Gündüz Ur (2015). Schwannoma of the sigmoid colon. BMJ Case Rep.

[10] Fletcher CD, Berman JJ, Corless C (2002). Diagnosis of gastrointestinal stromal tumors: a consensus approach. Hum Pathol.

[11] Kim HJ, Kim CH, Lim SW, Huh JW, Kim YJ, Kim HR (2012). Schwannoma of ascending colon treated by laparoscopic right hemicolectomy. World J Surg Oncol.

[12] González Ruiz Y, Reyes Delgado A, Gutiérrez Alonso C, Franco Rubio JI, González Herrero M (2019). Sigmoid intussusception as a clinical presentation of colonic schwannoma: Pediatric case [Article in Spanish]. Arch Argent Pediatr.

